# Settings and Clinical Applications of Subthreshold Micropulse Laser Therapy: A Review

**DOI:** 10.3390/jcm13195729

**Published:** 2024-09-26

**Authors:** Tania Sorrentino, Davide Allegrini, Giacomo De Rosa, Francesco Santoru, Lorenzo Crepaldi, Alessandro Feo, Giacomo Zanellati, Stefania Marconi, Ferdinando Auricchio, Mario R. Romano

**Affiliations:** 1 ⁠Department of Biomedical Sciences, Humanitas University, Via Rita Levi, Montalcini 4, Pieve Emanuele, 20072 Milan, Italy; 2 Eye Center, Humanitas Gavazzeni-Castelli, 24128 Bergamo, Italy; 3 Department of Civil Engineering and Architecture, University of Pavia, Via Ferrata 3, 27100 Pavia, Italy; 4 Fondazione IRCCS Policlinico San Matteo, Viale Camillo Golgi 19, 27100 Pavia, Italy

**Keywords:** subthreshold, micropulse, laser, retina, settings, macular edema

## Abstract

Subthreshold lasers operate below the threshold of visible tissue damage, thereby preventing ophthalmoscopically visible thermal damage to the chorio-retinal layers. They could represent a safe and effective alternative and/or adjunctive procedure to conventional lasers in treating diabetic macula edema (DME), central serous chorioretinopathy (CSCR), and branch retina vein occlusion (BRVO). This review focuses on the use of subthreshold micropulse laser (SMPL), its settings, and clinical applications. Despite their widespread use, a standardized protocol for sub-threshold laser settings has not been established yet, and thus, there is uncertainty in selecting effective and safe parameters for any specific situation. We conducted a comprehensive overview of the existing indications for subthreshold laser therapy and their settings for different retinal diseases. The debate revolves around which parameters could guarantee the safety of the procedure for each case, depending on the duty cycle, the laser wavelength, the spot duration, and the power, with laser power titration on one side or choosing a fixed lowered power value on the other side. SMPL therapy for DME, CSCR, and BRVO-associated macular edema has shown significant effectiveness in reducing the macular thickness, facilitating subretinal fluid absorptions, increasing the best corrected visual acuity (BCVA) and reducing the number of intravitreal injections (IVI) required annually. We presented a broad list of the laser parameters reported in the literature, organized into different tables divided based on the specific pathology, with the aim of providing a useful tool for future studies.

## 1. Introduction

Traditional retinal laser treatments work on the application of supra-threshold energy, resulting in visible retinal tissue damage and potential side effects. Subthreshold laser treatments, which operate below the threshold for ophthalmoscopically visible tissue damage, represent a precise and gentle alternative to conventional lasers [1,2]. The use of a series of short, repetitive laser pulses allows the tissue to cool down between pulses, helping to prevent thermal damage, as the inner retina remains below the threshold of coagulative damage [2]. Among different types of subthreshold laser treatments, we focused our review on the subthreshold micropulse laser (SMPL), its settings, and clinical applications.

The rationale for using a subthreshold laser over a conventional one is that the former causes limited damage to the retinal and choroidal tissues. This allows for treatment in close proximity to the foveal area, permits multiple re-treatments over time, and avoids the known side effects associated with conventional laser treatment applied to the central macular region, such as central negative scotoma, post-laser unpredictable spot enlargement, and visual field loss [3].

A debated parameter for a successful subthreshold treatment is the appropriate laser power, as no standardized protocols for SMPL have been proposed, and there is significant variability in the choice of parameters, such as laser power, titration, duty cycle (DC), and pulse duration. 

This approach proves to be valuable in addressing conditions in which RPE stimulation could reduce the severity of intraretinal or subretinal fluid retention, including post-surgical inflammatory macular edema, diabetic macular edema, central serous chorioretinopathy, and retinal vascular diseases. It provides therapeutic benefits through the thermal stress response of the retina pigment epithelium (RPE) induced by the subthreshold laser treatment [4].

## 2. Methods

We conducted a comprehensive analysis of the relevant articles available in the literature up to the end of 2023. The information used to write this paper was collected from several sources, including Medline, the references of retrieved articles, and authoritative texts, in order to present a broad perspective on the topic and to provide our recommendations. We carried out a preliminary search of the literature to select articles containing the parameters of interest for our research, such as subthreshold laser setting values and clinical outcomes, including the central macular thickness (CMT) and the best corrected visual acuity (BCVA). The results of 144 articles were analyzed, through which we constructed 3 tables summarizing the inclusion criteria, the type of treatment performed, and the clinical outcomes obtained for each pathology. 

## 3. Subthreshold Laser Therapy

Subthreshold laser (STL) photocoagulation aims to induce therapeutic effects without visible intra-retinal damage targeting the RPE selectively and minimizing the negative thermal effects on the neural retina [5,6,7]. It activates a sequence of biological reactions, including upregulation and downregulation of RPE-mediated factors, cellular responses, inflammation modulation, tissue repair, and enhanced function of intra-RPE heat-shock proteins (HSPs), including HSP70 [5,7,8,9].

To achieve a less-damaging subthreshold treatment, it is crucial to create a moderate sub-lethal thermal elevation confined to the RPE cells, controlling the thermally affected volume and factors like retinal spot size, irradiance, and laser pulse duration [5,10].

Subthreshold laser treatments encompass a spectrum of photocoagulation techniques that provide therapeutic benefits for retinal or macular diseases while avoiding damaging laser scars in the treated area [3,11] These treatments were developed to preserve the RPE while effectively treating underlying macular pathology [6,11,12].

Five distinct types of subthreshold laser techniques have been identified [2,13].

These modalities exhibit fundamental differences in their underlying mechanisms, contributing to variations in their therapeutic effects.

1. A subthreshold micropulse laser (SMPL) achieves subthreshold effects by delivering laser energy through repetitive short pulses interspersed with periods of non-firing, as opposed to a continuous wave. In an in vitro study, sublethal photothermal stimulation with SMPL demonstrated the ability to stimulate the repair of the inner blood–retinal barrier and increase the activity of RPE without causing damage to photoreceptors [11,13];
2. Endpoint management (EpM) employs a computational model of retinal photothermal damage to minimize tissue damage. The protocol adjusts the laser’s power and duration by analyzing the visible threshold, aiming to achieve subthreshold effects. It is important to consider that EPM is still a form of titration algorithm, and therefore, greater care must be taken to avoid unintentional LIRD [14];
3. Selective retina therapy (SRT) utilizes short pulses on the order of microseconds to target RPE cells while sparing surrounding tissues, restoring the function of the photoreceptors. Achieved through high-energy pulses with very short durations (<5 microseconds), SRT selectively destroys damaged RPE cells and stimulates wound healing, referred to as “retinal rejuvenation” [11];
4. A Subthreshold nanosecond Laser (SNL) employs ultra-short pulses on the order of nanoseconds to specifically target RPE cells and spare surrounding tissues. Using a green wavelength to target melanosomes, SNL’s hypothesized mechanism is akin to that of SRT. Targeted elimination of RPE through microbubble formation leads to “retinal rejuvenation”, with surrounding healthy RPE filling the lasered tissue [15,16,17,18];
5. Transpupillary Thermotherapy (TTT) is a subthreshold laser technique utilizing a long-pulse, low-irradiance, and infrared photocoagulation laser [19]. In contrast to previous techniques, TTT is applied over a large spot, ranging between 0.5 and 3.0 mm, with a pulse duration of 1 min using an 810 nm near-infrared laser to minimize damage to the nerve fiber layer [20].

We focused on the SMPL laser, as it is the one with which we have the most experience, being an integral part of our daily clinical practice. Additionally, there is extensive literature available, enabling us to conduct our review with solid data.

## 4. Subthreshold Micropulse Laser Therapy

Subthreshold micropulse laser (SMPL) therapy involves delivering laser energy in a pulsed manner, allowing for intermittent energy release. This approach proves valuable for addressing conditions like macular edema and retinal vascular diseases, delivering therapeutic benefits while minimizing thermal damage [3,5,10]. The primary objective of subthreshold micropulse treatment protocols is to spatially confine damaging thermal elevations, thereby minimizing collateral damage. Unlike conventional threshold photocoagulation, where the final burn size exceeds the laser spot on the retina, micropulse treatment limits both axial and lateral spread, maintaining the treated RPE area close to the laser spot size [3,5,7,10].

This less-destructive laser therapy presents a more favorable benefit-to-risk ratio, justifying earlier treatment in the disease course and enabling stabilization or improvement of less compromised visual functions [8,21].

Importantly, the therapeutic effect of subthreshold micropulse laser treatment occurs without inducing ophthalmoscopically visible thermal damage to the retina, making it particularly advantageous for treatments near the fovea [12].

### 4.1. Tissue Interaction

SMPL employs a train of very short pulses to deliver energy to a single spot. The total amount of energy is intentionally limited to prevent tissue damage. However, this energy is sufficient to stimulate the RPE cells efficiently [22,23].

As a result, this method positively influences retinal biological processes, offering benefits such as the reduction of inflammation and the preservation of natural homeostasis within the glial cell population [3,24].

SMPL has been demonstrated, particularly in recent human studies, to downregulate various local factors, including vascular endothelial growth factor (VEGF), VEGF inducers, and permeability factors [2]. Experiments conducted on animals have shown that the expression of HSPs, primarily the HSP70, after SMPL irradiation is not significantly different from the more damaging conventional laser treatments involving RPE cell damage [25]. The HSP70 upregulates the antiapoptotic protein BCL2 and prevents the formation of the caspase-dependent apoptosis complex [25]. 

The induction of HSPs by subthreshold stimulus confirms that destructive irradiation is not necessary to evoke a biological response in the RPE layer [26].

Moreover, the RPE cellular healing response subsequent to laser stimulus reduces the expression of genes encoding for inflammatory mediators such as NFkB and TNF-alpha [25].

In vitro models, using mouse RPE cells, have been proposed to study the effects of a subthreshold micropulse laser on the RPE. A significant study conducted by Li Z. et al. involved irradiating mouse-derived RPE cells with an A 810 nm micropulse laser, setting a 5% duty cycle and the power between 100 and 400 mW. The results showed a decrease in the mRNA expression of angiogenic stimulators (VEGF-A, TGF-B, and bFGF), while the expression of the inhibitors (PEDF) significantly increased. Regarding cell viability, the same in vitro model demonstrated that, at a power of 100 and 200 mW with a 5% duty cycle, the 810 nm laser-induced apoptosis was at 3.52% and 3.55% respectively. However, at powers of 300 and 400 mW, with the same duty cycle of 5% and the same 810 nm laser, the apoptotic rates significantly increased at 9.31% and 14.24%, respectively [21].

A recent study suggests that there is a significant reduction in the VEGF concentration after SMPL treatment at both 3 and 12 months in eyes affected by diabetic macular edema (DME) compared to the baseline, particularly in comparison to healthy eyes [24].

Moreover, the same study observed changes in the expression of proteins associated with glial cells, including glial fibrillary acidic protein (GFAP) and inwardly rectifying potassium (Kir) 4.1, in patients with di (DME) treated with SMPL [24].

GFAP, an intermediate filament protein, is prominently expressed in activated retinal Müller cells. The decrease in GFAP levels implies a potential modulation of Müller cell activity in response to SMPL treatment [27].

Kir 4.1, found in Müller cells, plays a crucial role in regulating potassium conductance and significantly influences simultaneous water transport across cellular membranes [24].

The glial cell family includes Müller cells, astrocytes (macroglia), and microglial cells, playing a dual role in maintaining the structural integrity and homeostasis of the retinal environment [28].

Activated by stress conditions, such as chronic hyperglycemia, these glial cells can undergo morphological and functional changes. In instances of activation, as seen in conditions like diabetes, microglial cells migrate from the inner to the outer retina. Subsequently, they release pro-inflammatory and vasoactive substances, including VEGF. This contributes to a local inflammatory response, leading to heightened vascular permeability [28].

It has been proposed that microglial cells in the retina can be visualized using SD-OCT as hyperreflective foci (HRF) [29]. 

Recently, SMPL has been shown to reduce HRF, even at a long-term follow-up of 1 year. This reduction is accompanied by improvements in various parameters, including a decrease in the area of cysts, the reorganization of inner retinal layers, and a reduction in the number of microaneurysms [30].

The concentration of GFAP has been demonstrated to decrease at 12 months after the first SMPL application in eyes with DME, indicating the normalization of Müller cell activity. Similarly, the expression of Kir 4.1, associated with Müller cells, also decreases [31].

The decreased expression of both GFAP and Kir 4.1 suggests that SMPL may contribute to reducing the concentration of inflammatory cascade proteins produced by retinal glial cells [31].

### 4.2. Safety

The feared complications of conventional laser treatments (CLT) historically include visual decline and the development of scotomas due to chorioretinal damage, expanding spot scars, and subretinal fibrosis. This set of events is classically referred to as laser-induced retinal damage (LIRD) [32].

In contrast, SMPL is generally considered safe, and cases of overtreatment are rarely reported. Due to the specific characteristics of the SMPL parameters discussed above, there is no evidence of photoreceptor or RPE damage following subthreshold laser therapy, as demonstrated by ophthalmoscopy and imaging techniques, including optical coherence tomography (OCT), fundus autofluorescence (FAF), dye-based angiographic imaging, and biomicroscopy [2,5,7,33,34].

Additionally, in cases of insufficient or partial response, SMPL therapy may be repeated with a specific timing that varies depending on the type of pathology treated. In the case of DME, SMPL can be repeated after a minimum of 3 months from the previous treatment [35].

Ultimately SMPL treatment can be safely completed in a single session due to its safety profile and the absence of adverse effects or pain.

## 5. Settings of SMPL

The micro-pulsed mode of delivering energy to the retina, with specific duty cycle settings, subthreshold power settings, and spot duration, can be applied to various lasers currently available on the market that operate at different wavelengths, including 810 nm (infrared), 532 nm (green), 577 nm (yellow) and 670 nm (red) [36]. 

Relevant parameters for SMPL include duty cycle (DC), laser power, titration, exposure time, and spot size [2,5,7,33,34]. SMPL technology typically uses higher power compared to the power values used in conventional continuous-wave (CW) photocoagulation. However, the final irradiance is lower because it is reduced by the limited duty cycle to avoid any unintended laser-induced retinal damage (LIRD) to the RPE and neurosensory retina.

The data presented in the tables below primarily detail the parameters used for the two types of lasers, namely the infrared at 810 nm and the yellow at 577 nm, which are those frequently used in the study analyzed in this review.

### 5.1. Laser Power 

There is uncertainty in choosing the appropriate laser power settings for SMPL therapy [37]. Various therapeutic approaches exist, including laser power titration and setting power to fixed low values.

Titration involves a gradual increase in power, typically performed on the border of the edematous retina, until a threshold value is reached [3,5,10]. Once the laser impact is barely visible as retinal whitening, 30–50% of the threshold value may be used [36]. The power is carefully titrated to ensure that the temperature in the targeted tissue remains confined to sub-lethal levels, thereby avoiding the production of any visible lesion (subvisible threshold) [3,5].

This process must be carefully executed to balance the risk of overtreatment and LIRD, which is more likely using a 577 nm wavelength laser compared to an 810 nm laser and could lead to visible burns on the retina on one hand, and undertreatment which could lead to treatment failure on the other [9,12].

However, titration is not considered a standardized procedure. First, it is challenging to determine the specific area of the retina where it should be performed, and second, there is no clear guidance on how much to reduce the laser power once the threshold value is found [36]. For these reasons, the International Retinal Laser Society (LIGHT) suggests using fixed laser parameters (the same settings in all eyes) to obtain the therapeutic effect and reduce the likelihood of unintended retinal damage [3,37].

Donati and colleagues were the first to study the morpho-functional outcomes of patients affected by mild center-involving DME treated with two different settings of yellow 577 nm SMPL, using a fixed or a variable regimen delivered with the same DC (5%) [38]. They showed that both approaches were effective in terms of visual stabilization and reduction of central retinal thickness. However, fixed SMPL (F-SMPL) treatment appeared more suitable compared to variable SMPL (V-SMPL), minimizing treatment time and reducing the possible errors associated with incorrect titration when switching from continuous to micropulse mode [38].

Given the current uncertainty in setting these parameters, prospective, large, randomized, controlled studies are necessary to further understand the effectiveness and reliability of SMPL when utilizing different parameters and treatment regimens.

### 5.2. Duty Cycle

The duty cycle (DC) is another important SMPL parameter that influences the amount of energy delivered to the retinal tissues and the potential for thermal damage. DC is defined as the ratio of the laser’s “on” time to its total cycle time and is expressed as a percentage. Common DCs for SMPL therapy range from 5% to 15% [5,7,33,34].

Luttrull et al. found that a DC higher than 5% with an 810 nm subthreshold laser was associated with an increased risk of retinal burn, and this risk increases further when using shorter wavelength lasers [39].

In a study conducted by Yu et al. on enucleated rabbits’ eyes, of the eyes treated using an 810 nm micropulse laser or a 532 nm micropulse laser with 5%, 10%, 20%, and 40% DC, only the 5% DC therapy caused no retinal damage [40].

Chhablani et al. described the yellow subthreshold microsecond laser as safe and effective with both 5 and 15% DCs following careful titration compared to a CW laser in DME [41]. However, even though the 15% DC setting seemed to achieve better functional outcomes and the largest decrease in volume of the subretinal fluid in CSCR, they found ophthalmoscopically visible burns in 1 out of 10 eyes treated with 15% DC [41].

Indeed, several studies described retinal burns in SMPLs performed with 10% and 15% duty cycles [3,17,22,42].

### 5.3. Exposure Time and Pulse Duration

In SMPL therapy, the laser energy is delivered in a micropulse fashion with alternating “on and off” periods. More specifically, a series of repeated brief laser pulses distribute laser energy within an “envelope” with a standard width of 0.1 to 0.5 s (exposure time). The duration of each individual laser pulse (pulse duration) is usually in the range of 100–300 ms. The “envelope” consists in both “off” time, which is the interval between the micropulses, and “on” time, which is the length of every micropulse. The “off” duration is crucial because it allows the cooling process between pulses. The time interval (T) is equal to the sum of the “on” and “off” time, and 1/T represents the frequency (f), i.e., the pulse per second, expressed in Hertz (Hz) [5,7].

### 5.4. Spot Size

Since the SMPL targets the RPE, an extensive RPE area has to be treated to improve clinical outcomes [1,36].

LIGHT suggests avoiding focal treatment and instead performing panmacular treatment with confluent laser spots between the vascular arcades, covering the edematous retina and the foveal center [37].

Spot size is still, to this day, a parameter that causes confusion, as there is a significant variability in its choice across the studies we compared in the review. For a long time, it was chosen based on personal preferences and prior experience rather than on evidence, likely due to the past uncertainty about the mechanisms and biophysics of laser action.

In treating DME with SMPL, the spot size ranged between a minimum of 100 µm to a maximum of 210 µm, with a mean value of 145 µm. Across the studies presented in Table 1, a spot size of 100 µm was used in eight studies, making it the most commonly used, spots of 200 µm were used in seven studies making this size the second most commonly used one, and lastly, spot sizes of 125 µm, 160 µm, and 210 µm were used in two studies, four studies, and one study, respectively.

In treating CSCR with SMPL, spot size ranges between a minimum of 100 µm to a maximum of 200 µm with a mean value of 144 µm. Among the studies presented in Table 2, the most commonly used spot diameter was 160 µm, with nine studies choosing it. A spot size of 100 µm was used in eight studies, making it the second most commonly used size, and lastly, sizes of 112.5 µm, 125 µm, 150 µm, and 200 µm were used in one study, five studies, one study, and six studies, respectively.

In treating BRVO with SMPL, spot size ranges between a minimum of 100 µm to a maximum of 200 µm. As reported in Table 3, authors chose to use 100 µm or 200 µm as the diameter of the spot in two studies and, lastly, 125 µm in three studies.

## 6. Clinical Application

SMPL is considered an alternative treatment for macular disorders associated with macular edema [7,34].

A summary of the collected data and the most commonly used settings in the literature are presented in Table 1, Table 2 and Table 3 for macular edema associated with DME, CSCR, and BRVO, respectively.

### 6.1. Diabetic Macular Edema

The efficacy of SMPL versus conventional photocoagulation or intravitreal injections in the treatment of DME has been compared in three meta-analyses.

In 2016, Chen G. et al. analyzed the results of six randomized controlled trials (RCTs) including 398 eyes. They found similar anatomical outcomes and better visual acuity in the group treated with STL therapy compared to those treated with conventional laser photocoagulation [42].

In the same year, Qiao G. et al. analyzed the results of 425 eyes from seven different studies and found that SMPL showed an equal effect on visual acuity, contrast sensitivity, and reduction of DME compared to conventional mETDRS protocol, inducing less retinal damage [100]. In 2017, Wu Y. et al. performed a Bayesian network meta-analysis finding that there was no significant difference in functional outcomes between SMLP and CLT. However, the most effective treatment was Ranibizumab therapy combined with CLT followed by SMLP monotherapy, Bevacizumab therapy combined with CLT, and CLT monotherapy [101].

In 2023, Tai F. et al. performed a systematic review and meta-analysis, including fourteen RCTs comprising 514 eyes treated with a conventional laser and 574 eyes treated with a subthreshold laser. No difference in functional outcomes and rates of adverse events at 12 months were found. A small reduction in central retinal thickness, which is unlikely to be clinically significant, was observed in the group treated with a conventional laser [102].

Recently, Hu X. et al. analyzed eight randomized controlled trials involving a total of 546 eyes for comparison in a meta-analysis of SMLP to CLT in the treatment of DME. They found that SMLP, compared with CLT, could have superior efficacy and safety in the improvement of BCVA, reduction of CMT, and preservation of contrast sensitivity [103].

By using angio-OCT, along with the improvement in functional and anatomical outcomes in naïve DME treated with STL, Li G. et al. studied several microvascular perfusion parameters, including vessel density, vessel length density, and fractal dimension. They found an improvement in the deep capillary plexus and choriocapillary plexus at 6 months post-STL treatment [104].

The DIAMONDS (diabetic macular edema and diode subthreshold micropulse laser) trial compared CLT with SMPL to treat diabetic macular edema suitable for a macular laser (CMT < 400 microns), finding no differences in anatomical and functional outcomes, even with a slightly higher number of laser treatments in the STL group [105]. According to the findings from these meta-analyses, SMPL could represent a valid alternative to conventional laser treatment and a solid adjuvant to an intravitreal therapy regimen for DME.

SMPL could be introduced as a first-line therapy for treating DME, in adjunction to the gold-standard IVI regimen, to stabilize the anatomical and functional outcomes and to reduce the number of injections required per year.

### 6.2. CSCR

Subthreshold lasers are of great interest in the treatment of CSCR due to the lack of available effective treatments and because it is a condition that affects the central region of the macula. Conventional laser treatments in this area are associated with several side effects, making subthreshold laser therapy a more attractive option.

In 2008, Lanzetta et al. described in a pilot study the efficacy of nonvisible micropulse diode laser irradiation in the treatment of CSCR, showing improved metabolic function of RPE cells and their capability to drain the subretinal fluid, which is the mechanism that could lead to the resolution of visual impairment [1].

Wu Z. et al. analyzed four RCTs and five retrospective studies with 790 eyes with chronic CSRC, finding that SML significantly improved the best-corrected visual acuity (BCVA) compared with PDT at 6 to 8 weeks, 6 months, and 7 to 8 months in patients with chronic CSCR [106].

Li X. et al. in a meta-analysis included eleven studies with 834 eyes, where 428 eyes underwent SML treatment and 406 eyes received other interventions. They found that the clinical efficacy of SML therapy was similar to other treatments without serious side effects [107].

Although spontaneous healing of some patients with acute CSCR, Long He et al. described that the therapy with 577 nm SML can shorten the disease course, reducing the risk of chronic transformation and improving visual acuity [67].

Zhou L. et al. compared the SML therapy with the CLT in acute CSCR, finding that 577 nm SML has the same effectiveness as 577 nm CL for improving anatomical and functional outcomes at 6 months with less damage to the retina [69].

Altınel MG et al. analyzed the optical coherence tomography parameter predictors of treatment response to a 577 nm SMLT in chronic CSRC, finding that the presence of baseline intact EZ and RPE and extrafoveal foci can potentially be used as predictors of the SML treatment success in chronic CSCR [75].

Across the studies analyzed in the review, SMPL has demonstrated a significant reduction in central macula thickness (CMT), a significant improvement in BCVA, and may reduce the recurrence of the visual impairment [69]. Due to its high safety profile, repeatability, and absence of visual side effects, SMPL could be introduced as a first-line interventional therapy for CSCR. It may be used in the initial presentation of the condition to reduce the risk of developing a chronic form and long-term visual impairment.

### 6.3. BRVO

Despite anti-VEGF therapies and traditional retinal laser photocoagulation being considered effective treatments for BRVO-CME, they are not without risks or side effects, and new treatment options have been proposed [94]. The application of 810 nm diode SMPL for BRVO-CME was first described in 1997 by Friberg et al., while recently, 577 nm SMPL has been reported to be effective against BRVO-CME, with a significant therapeutic effect but without visible retinal damages [99,108].

Parodi et al. compared the use of conventional laser treatment and SMPL in BRVO-CME and found that the resolution of macular edema and the visual acuity improvement were similar, but SMPL did not determine retinal biomicroscopic or angiographic visible signs [99].

Ozkurt et al. did not find a significant difference between anti-VEGF Ranibizumab and SMPL in reducing macular thickness and increasing visual acuity at 1-year follow-up, as both treatments were shown to be beneficial [95]. Terashima et al. highlighted how the combination of Ranibizumab intravitreal injection (IVI) and SMPL is not significantly different from IVI alone for improving VA and reducing macular thickness but reduces the number of IVI needed [94].

In a prospective randomized clinical trial, Parodi et al. evaluated the use of SMPL versus bevacizumab IVI in BRVO-associated macular edema resistant to traditional laser photocoagulation. At 1-year follow-up, bevacizumab IVI demonstrated significant effects on BCVA and central foveal thickness (CFT), while SMPL did not show beneficial activity [96].

In accordance with the analyzed data in the review, we conclude that SMPL should not be used as a stand-alone therapy for treating BRVO-CME, but it could be combined with the gold-standard IVI regimen, potentially enhancing the effects of anti-VEGF by further reducing the CMT and decreasing the number of IV injections required annually.

## 7. Conclusions

Subthreshold micropulse laser treatment has demonstrated safety and efficacy in the management of diabetic macular edema (DME), CSCR, and macular edema secondary to venous occlusion. Despite SMPL’s increasing use in clinical practice, significant variability remains in the selection of laser parameters among practitioners, highlighting ongoing challenges and a need for standardized protocols. For instance, some practitioners adjust the power in micropulse mode based on titration, often dividing the value by two, which may extend the duration and expose patients to the risk of more side effects. Other practitioners multiplied the threshold value by 0.5–4 when switching from the continuous wave to the micropulse mode. Other practitioners use fixed values that consist of the same identical settings in all eyes. Moreover, the International Retinal Laser Society (LIGHT) suggests using fixed parameters, discouraging the titration mode due to the risk of unintentional LIRD [37].

Considering this, it is clear that there is a great variability in preferences and recommendations among different authors and practitioners.

The variability in protocols highlights the critical need for a more precise and comprehensive understanding of the optimal laser parameters for SMPL treatments. This variability in treatment outcomes arises from practitioners lacking sufficient knowledge of new scientifically validated principles of safe and effective subthreshold laser treatment. Specifically, new evidence suggests that practitioners should use the 810 nm wavelength laser with a duty cycle of 5% over the 577 nm wavelength one, as it is difficult, if not impossible, to cause any LIRD with an 810 nm wavelength laser at 5% DC, while it is more likely to cause unintentional retinal damage using the 577 nm, even at a 5% DC and low power values. It is also advisable to not exceed a 5% duty cycle for macular treatments and to prefer a confluent high-density application over extensive retinal areas [9]. The lack of standardization across studies can lead to inconsistent outcomes, making it challenging for practitioners to determine the most effective approaches. This review, therefore, aims to be a significant resource for clinicians and researchers by thoroughly summarizing the types of lasers used, the specific parameters applied in various studies, and the clinical outcomes observed over time. By consolidating this diverse information, the review not only aims to enhance the learning process for practitioners but also provides a solid foundation for guiding future research. Additionally, it seeks to contribute to refining the treatment protocols. This approach is intended to support the development of standardized practices, leading to improved patient outcomes and advancing the field of SMPL treatments.

## Figures and Tables

**Table 1:** SMPL therapy in diabetic macular edema.

First Author	Year	Study Design	Eyes (N=)	Inclusion Criteria	Laser Type	Spot Diameter	Duty Cycle	Power	Safety	BCVA	CMT	FU (Months)
(Zavorkova et al., 2023) [43]	2023	Prospective	52	DR complicated by DME	577 nm MicroPulse yellow diode laser	100 μm	5%	Continuous laser power was titrated to a barely visible burn, then switched to MicroPulse mod with power 2–3 times bigger than that measured during dosing. (between 400 and 700 mW)	No side effects	No statistically significant difference (*p* > 0.05) T0 70.0, SD 10.1 ETDRS 1y 72, SD 10.0 letters 5y 66.9, SD 12.1 ETDRS	Decreased (*p* < 0.05) T0: 345.9 µm SD 122.6 5 years: decreased by 89.5 µm SD 153.6	60
(Tatsumi et al., 2022) [44]	2022	Prospective	51	DME with central involvement CMT ≥ 300 μm Baseline BCVA from 0.7 to 0.05	577 nm Yellow	200 μm	10%	A test burn was made and a subthreshold power was determined by titrating the burn to be barely visible (120–170 mW)	No side effects	No statistically significant difference (*p* > 0.05) T0: 0.478 ± 0.320 logMAR 48 w: 0.279 ± 0.222 logMAR 96 w 0.283 ± 0.273 logMAR	No statistically significant difference (*p* > 0.05) T0: 472.8 ± 136.1 µm, T48w 344.7 ± 73.1 µm 96w 329.0 ± 78.5 μm	24
(Kikushima et al., 2021) [45]	2021	Retrospective	43	Treatment-naïve DME or DME not treated within the last 4 months; CRT > 200 μm; BCVA > 0.05	577 nm Yellow 670 nm Red	210 μm	10%	Power was increased until a visible mark on the retina, then reduced (120–350 mW)	No side effects	No statistically significant difference (*p* > 0.05) Yellow SMPL group: T0: 0.40 ± 0.33 LogMAR, 12m: 0.45 ± 0.35 LogMAR Red SMPL group: T0: 0.35 ± 0.22 LogMAR 12 m 0.33 ± 0.28 LogMar	Decreased (*p* < 0.05) yellow SMPL group T0: 449 ± 169 12 m: 389 ± 159 µm red SMPL group T0: 515 ± 171 µm 12 m: 415 ± 196 µm	12
(Donati et al., 2021) [38]	2021	Retrospective	39	Type 1 or 2 DM and HbA1c < 10%; CRT ≤ 400 mm, (BCVA) ≥ 35 letters on ETDRS chart	577 nm Yellow	100 μm	5%	Continuous laser power was titrated to a barely visible burn, then switched to MicroPulse mode, multiplying the test burn power by 4 250 mW	No side effects	No statistically significant difference (*p* > 0.05) T0: 0.297 ± 0.431 LogMAR 12 m: 0.289 ± 0.473 LogMAR	Decreased (*p* < 0.05) T0: 371.06 ± 37.8 μm 12 m: 325.60 ± 110.0 μm	12
(El matri et al., 2021) [46]	2021	Retrospective	98	Treatment-naïve central DME with (BCVA) ≥ 20/400, CMT ≤ 500 μm, HbA1C < 9%	577 nm Yellow	200 μm	5%	400 mW	No side effects	Improved (*p* < 0.05) T0: 0.692 ± 0.35 LogMAR 48 w: 0.501 ± 0.37 LogMAR	Decreased (*p* < 0.05) T0: 479.1 ± 14.3 μm 48 w: 289.6 ± 15 μm	12
(Passos et al., 2021) [47]	2021	Retrospective	56	Type I or II DM; center-involving DME; BCVA ≤ 20/40;	577 nm Yellow	160 μm	5%	Power titrated to the thermal threshold, in an area of non-edematous retina, then reduced by 50%.	No side effects	Improved (*p* < 0.05) T0: 0.59 ± 0.32 LogMAR 14 w: 0.43 ± 0.25 LogMAR	No statistically significant difference (*p* > 0.05)	14
(Citirik, 2021) [48]	2021	Retrospective	70	Diabetic patients with recurrent macular edema previously treated with 3 or 4 monthly consecutive intravitreal Ranibizumab injections.	577 nm Yellow	200 μm	-	Retinal burn power was titrated from 100 mW upward until a burn became visible., then reduced to 30%.	No side effects	Improved (*p* < 0.05) (Group1)T0: 0.54 ± 0.05 (Group1)2 w: 0.39 ± 0.06	Decreased (*p* < 0.05) 272.48 ± 39.45 μm 229.64 ± 38.68 μm	2
(Frizziero et al., 2021) [49]	2021	Retrospective	134	Type 1 or 2 DM with HbA1C < 8%; DME previously untreated with CRT ≤ 400 μm	577 nm Yellow	100 μm	5%	250 mW	No side effects	Improved (*p* < 0.05) Baseline 77.3 ± 4.5 ETDRS End 79.4 ± 4.4 ETDRS	Decreased (*p* < 0.05) Baseline 327.2 ± 45.8 μm End 324.9 ± 27.2 μm	16.6 ± 6.5
(Lai et al., 2021) [50]	2021	Retrospective	164	Patients with type 1 or 2 DM; CMT > 300 μm; BCVA between 20/30 and 20/400	577 nm Yellow	200 μm	5%	400 mW	No side effects	Improved (*p* < 0.05) visual acuity change from baseline at 6 months: 0.077 ± 0.237 visual acuity change from baseline at 24 months: 0.132 ± 0.280	Decreased (*p* < 0.05) Central macular thickness change from baseline at 6 months: μm 44.5 ± 81.1 Central macular thickness change from baseline at 24 months: μm 112.1 ± 100.5	24
(Filloy et al., 2020) [51]	2020	Retrospective	23	OCT-confirmed CI-DME; patients asymptomatic with BCVA ≥ 20/32	577 nm Yellow	160 μm	5%	1/3 of the minimum power whitened the retina in the macular periphery.	No side effects	No statistically significant difference (*p* > 0.05)	Decreased (*p* < 0.05) mean reduction of 16.9 μm at 12 w mean reduction of 22 μm at the end of the follow-up	Up to 18
(Vujosevic et al., 2020) [52]	2020	Prospective	37	No previous treatment for DME; CMT ≤ 400 μm; BCVA ≥ 78 letters (ETDRS) score, or BCVA < 78 letters ETDRS score who refused or had contraindications for Anti-VEGF intravitreal injection treatment.	577 nm Yellow	100 μm	5%	250 mW	No side effects	Improved (*p* < 0.05) T0: 69.4 ± 12.0 ETDRS letters 12 m: 76.0 ± 9.1 ETDRS letters	No statistically significant difference (*p* > 0.05) T0: 304.95 ± 50.69 μm 12 m: 294.49 ± 39.8 μm	12
(Kanar et al., 2020) [53]	2020	Prospective	28	Type 2 DM with DME center involving; BCVA from 0.2 to 0.9 (Snellen); CMT ≥ 300 μm; no ischemic maculopathy on FA	577 nm Yellow	160 μm	5%	Laser Power titrated to the retinal thermal threshold for each patient, then reduced by 50%.	No side effects	Improved (*p* < 0.05) T0: 0.40 ± 0.09 LogMAR 12 m: 0.17 ± 0.06 logMAR	Decreased (*p* < 0.05) T0: 466.07 ± 71.79 µm 12 m: 312.0 ± 39.29 µm	12
(Citirik, 2019) [54]	2019	Prospective	80	Recurrent DME, defined as CFT > 250 μm, previously treated with Ranibizumab	577 nm Yellow	160 μm	5%	Laser power titrated to the retinal thermal threshold for each patient, then reduced by 50%.	No side effects	Improved (*p* < 0.05) (Group 1) baseline 0.52 ± 0.05 LogMAR (Group 1) 2 months follow-up 0.38 ± 0.04 LogMAR	Decreased (*p* < 0.05) (Group 1) baseline 276.00 ± 22.44 µm (Group 1) 2 months follow-up 238.57 ± 25.87 µm	6
(Hamada et al., 2018) [14]	2018	Prospective	10	Type 2 diabetes with macular edema involving the fovea, active leakage on FA, CMT ≥ 300 μm; BCVA 0.3–1.0 logMAR; contraindication for direct microaneurysm photocoagulation ≥ 12 weeks	577 nm Yellow	200 μm	5%	Laser power titrated to the retinal thermal threshold for each patient, then reduced by 50%.	No side effects	No statistically significant difference (*p* > 0.05)	Decreased (*p* < 0.05) T0: mean 499.0 μm 6 M mean 337.6 μm	6
(Moisseiev et al., 2018) [55]	2018	Retrospective	38	Patients treated for central DME with anti-VEGF injections and/or micropulse laser; no more than 3 prior intravitreal injections of Ranibizumab, last injection > 2 months before	577 nm Yellow	100 μm	5%	400 mW	No side effects	Improved (*p* < 0.05) T0: 0.29 ± 0.12 logMAR 12 m: to 0.24 ± 0.17 logMAR	No statistically significant difference (*p* > 0.05) T0: 316.8 ± 91.5 µm 12 m: 282.6 ± 59.1 µm	12
(Latalska et al., 2017) [56]	2017	Prospective	75	Diabetic patients qualified for micropulse laser if they did not agree to start or continue anti-VEGF treatment for DME.	577 nm Yellow	100 μm	5%	Power titrated to the thermal threshold, then laser switched into micropulse mode and power doubled	No side effects	No statistically significant difference (*p* > 0.05) baseline median BCVA: 0.20 ± 0.27 LogMAR 6 months median BCVA: 0.30 ± 0.37 LogMAR	Decreased (*p* < 0.05) Baseline median CMT: 500 ± 205 µm 6 months median CMT: 346 ± 153 µm	6
(Elhamid, 2017) [57]	2017	Prospective	20	DME CMT ≥ 300 um; BCVA 0.1–0.8. Persistent DME after ≥3 IVI of anti-VEGF with last injection performed at least 3 months before potential Ozurdex injection	577 nm Yellow	200 μm	5%	400 mW	No side effects	Improved (*p* < 0.05) T0: 0.45± 0.14 Snellen 12 m 0.59 ± 0.14 Snellen	Decreased (*p* < 0.05) T0: 420.7 ± 38.74 µm 12 m: 285.2 ± 14.99 µm	12
(Vujosevic et al., 2015) [58]	2015	Prospective	53	Type 1 or 2 DM and an HbA1C ≤ 10%, previously untreated center-involving macular edema with CMT up to 400 µm BCVA of at least 35 letters on the modified ETDRS chart	577 nm yellow light laser 810 nm diode laser	100 µm 125 µm	5% 5%	250 mW 750 mW	No side effects	No statistically significant difference (*p* > 0.05) T0: 79.7 ± 6.1 ETDRS Letters 6 m: 78.7 ± 7.4 ETDRS Letters	No statistically significant difference (*p* > 0.05) T0: 357.8 ± 46.1 µm 6 m: 339.9 ± 55.7 µm	6
(Inagaki et al., 2015) [59]	2015	Prospective	53	Non-proliferative diabetic retinopathy or proliferative diabetic retinopathy with clinically significant macular edema (ETDRS criteria) involving either the center of the macular region or a border involving the foveal avascular zone. Diffuse dye leakage confirmed at FFA	810 nm MP laser 577 nm MP laser	200 µm	15%	Power titrated to the thermal threshold, then laser switched into micropulse mode and employed by 60% Mean energy 954.9 mW (500–2000 mW) in the 810 MP group 204.1 mW (180–400 mW) in the 577 nm MP group	No side effects	No statistically significant difference (*p* > 0.05) Baseline BCVA Infrared: 0.594 (SD 0.414) Yellow: 0.305 (SD 0.309) 12 months follow-up mean BCVA Infrared: 0.594 Logmar Yellow 0.275 Logmar	Decreased (*p* < 0.05) Baseline CMT Infrared 488.2 µm (SD 175.8) Yellow 416.9 µm (SD 112.9) 12 months follow-up mean CMT Infrared 361.8 µm Yellow 335.2 µm	12
(Kwon et al., 2014) [60]	2014	Retrospective	14	DM with recent history of visual deterioration; Cystic macular lesion on OCT and/or CMT > 260 μm.	577 nm Yellow	100 μm	15%	Power titrated to the thermal threshold, then laser switched into micropulse mode 100–180 mW	No side effects	Improved (*p* < 0.05) T0 0.51 ± 0.42 LogMAR Final follow-up 0.40 ± 0.35 logMAR	Decreased (*p* < 0.05) T0: 385.0 ± 111.0 μm Final follow-up 327.0 ± 87.7 μm	7.9 ± 1.6
(Figueira et al., 2009) [61]	2009	Prospective	84	Type 2 diabetic patients with CSMO (as defined by ETDRS study)	810 nm infrared diode laser	125 µm	15%	Power to achieve the visible laser burn doubled	Laser scars in 6/43 eyes treated	No statistically significant difference (*p* > 0.05) Baseline: 78.4 (SD 8.1) ETDRS letters 12 months: 75.0 (SD 13.7) ETDRS letters	No statistically significant difference (*p* > 0.05) Baseline:248.9 (SD 58.7) mm) 12 months: increase in CMT of 41.9 (SD 103.8)	12
(Laursen et al., 2004) [62]	2004	Prospective	23	Type I or type II DM Clinically significant macular edema HbA1c ≤ 10.0 Blood pressure (160/100 mm Hg	Subthreshold micropulse diode laser (814 nm)	125 µm	-	Power was initially adjusted upward to the minimum threshold value for a barely visible burn, then reduced to 50%	No side effects	No statistically significant difference (*p* > 0.05)	Decreased (*p* < 0.05)	6.5

**Table 2:** SMPL therapy in CSCR.

First Author	Year	Study Design	Eyes (N=)	Inclusion Criteria	Laser Type	Spot Diameter	Duty Cycle	Power	Safety	BCVA	CMT	FU (Months)	Additional Treatment
(Enrìquez-Fuentes et al., 2023) [63]	2023	Prospective	149	chronic CSCR with SRF > 4 months.	577 nm Yellow	150 μm	5% 10%	400 mW	Hyperplasia of the retinal pigment epithelium (HRPE)	Improved (*p* < 0.05) in 144 eyes; decreased (*p* < 0.05) in 5 HRPE eyes	-	2	
(Lee et al., 2023) [64]	2023	Retrospective	31	SRF involving the fovea ≥ 3 months on OCT scans; FU ≥ 6 months;	577 nm Yellow	200 μm	-	Laser Power titrated to the retinal thermal threshold for each patient; laser power was then reduced to 30%.	No side effects	No statistically significant difference (*p* > 0.05) T0: 0.31 ± 0.29 logMAR 6 m: 0.31 ± 0.40 logMAR	Decreased (*p* < 0.05) T0: 350.7 ± 112.76 µm 6 m: 239.71 ± 130.25 µm	6	
(Zeng et al., 2022) [65]	2022	Retrospective	18	Symptoms of CSC ≤ 3 months or less; SRF involving the fovea	810 nm diode laser	125 μm	15%	Laser Power titrated to the retinal thermal threshold for each patient, then switched to micropulse mode and power reduced	No side effects	Improved (*p* < 0.05) T0: 63.67 ± 9.79 (ETDRS) 12 m: 75.28 ± 12.58 (ETDRS)	Decreased (*p* < 0.05) T0: 427.28 ± 52.23 μm 12 m: 243.67 ± 39.46 μm	12	
(Kiraly et al., 2022) [66]	2022	Prospective	31	CSC < 3 months onset; no other retinal diseases	577 nm Yellow	-	5%	250 mW	No side effects	-	Decreased (*p* < 0.05)	6	
(Long et al., 2022) [67]	2022	Retrospective	34	CSC < 3 months, not previously treated; no other concomitant retinal disease	577 nm Yellow	200 μm	5%	Laser power titrated to the retinal thermal threshold for each patient; laser power was then obtained as a quadruple of the titration energy	No side effects	Improved (*p* < 0.05) T0: 0.48 ± 0.20 logMAR 6 m: 0.01 ± 0.06 lofMAR	Decreased (*p* < 0.05) T0: 485.38 ± 151.44 µm 6 m: 291.38 ± 26.46 µm	6	
(Chhablani et al., 2021) [68]	2021	Retrospective	101	Chronic or recurrent leakage in CSC (symptoms > 3 months) treated with micropulse laser and follow up ≥5 months after treatment.	577 nm Yellow	100–200 μm	Variable (5–15%)	Variable (170–866 mW)	No side effects	Improved (*p* < 0.05) T0: 0.35 ± 0.3 lgoMAR 10 m: 0.27 ± 0.31 logMAR	Decreased (*p* < 0.05) T0: 325 ± 130 µm 10 m: 255 ± 115 µm	10 (Range 5–36)	
(Zhou et al., 2021) [69]	2021	Prospective	55	CSC symptoms ≥ 4 weeks, leakage ≥ 300 μm away from fovea on FA; choroidal vessels dilatated on ICGA; SRF involving the fovea on OCT	577 nm Yellow	100 μm	5%	Laser power titrated to the retinal thermal threshold for each patient; laser power was then reduced by 50%	No side effects	Improved (*p* < 0.05) T0: 0.32 ± 0.21 logMAR 6 m: 0.00 ± 0.10 logMAR	Decreased (*p* < 0.05) T0: 474 ± 154 µm 6 m: 221 ± 74.4 µm	6	
(Altinel et al., 2021) [70]	2021	Retrospective	52	CSC with SRF involving macula center ≥ 6 months; at least 6 months of follow up; diffuse leakage on FA with corresponding hyperfluorescence on ICGA	577 nm Yellow	160 μm	5%	Laser power titrated to the retinal thermal threshold for each patient; laser power was then reduced by 50%	No side effects	Improved (*p* < 0.05)	Decreased (*p* < 0.05)	8.42 ± 3.34	
(Schworm et al., 2021) [71]	2021	Prospective	42	CSC, visual acuity score ≥ 35 on ETDRS charts. SRF ≥ 4 months, mottling of the RPE on OCT, active leakage on FA, and hyperflurescence on ICG	577 nm Yellow	200 μm	-	Laser power titrated to the retinal thermal threshold for each patient; laser power was then reduced to 30%.	No side effects	Improved (*p* < 0.05) T0: 79.7 ± 12.9 (ETDRS) 6 m: improved by 4.9 ± 8.6 ETDRS letters	Decreased (*p* < 0.05) T0: 398 ± 135 µm 6 m: 291 ± 68 µm	6	
(Uzlu et al., 2021) [72]	2021	Retrospective	20	Evidence of CSC on OCT; leakage at the RPE level on FA	577 nm Yellow	100 μm	5%	Laser power titrated to the retinal thermal threshold for each patient; laser power was then reduced by 50%. 160–200 mW	No side effects	Improved (*p* < 0.05) T0: 0.24 ± 0.28 logMAR 6 m: 0.18 ± 0.27 logMAR	Decreased (*p* < 0.05) T0: 308.10 ± 95.25 µm 6 m: 203.88 ± 72.79 µm	6	
(Prasuhn et al., 2021) [73]	2021	Prospective	27	CSC with SRF involving the macula on OCT and persistent SRF ≥ 3 months. Evidence of leakage on FA.	577 nm Yellow	200 μm	10%	Laser power titrated to the retinal thermal threshold for each patient; laser power was then reduced by 40–50%.	No side effects	No statistically significant difference (*p* > 0.05) T0: 0.4 logMAR (range 0.1–0.14) 4 w: 0.3 logMAR (range 0.1–0.5)	Decreased (*p* < 0.05) T0: 306 µm (range 254–389) 4 w: 266 µm (range 236–324)	1	
(Işik et al., 2020) [74]	2020	Retrospective	58	Symptomatic CSC ≥ 3 months; SRF involving the fovea as shown on OCT	577 nm Yellow	160 μm	5%	Laser power titrated to the retinal thermal threshold for each patient; laser power was then reduced by 50%.	No side effects	Improved (*p* < 0.05) T0: median BCVA 0.22 logMAR Final median BCVA: 0.0 logMAR	Decreased (*p* < 0.05) T0: 438 μm (mean: 455 μm) Final median CMT: 220 μm (mean: 243 μm)	11.4 ± 8.5	
(Guzin Altinel et al., n.d.) [75]	2020	Retrospective	39	CSC with SRF on the macula center ≥ 4 months; ≥6 months of follow-up; leakage area or irregular RPE defects on FA.	577 nm Yellow	160 μm	5%	Laser power titrated to the retinal thermal threshold for each patient; laser power was then reduced by 50%.	No side effects	No statistically significant difference (*p* > 0.05) T0: 0.26 ± SD 0.24 T12M: 0.25 ± SD 0.27	Decreased (*p* < 0.05) in remission group. Stable in failure group. (Remission) Baseline: 330.43 ± 105.48 (Remission) 12 Months 220.17 ± 72.37	24	
(Zhou et al., 2019) [76]	2019	Prospective	54	CSC duration < 6 months; no previous treatment for CSC	577 nm Yellow	100 μm	5%	Power titration started at 600 mW and increased until a visible burn. When this threshold was reached, the power was reduced by 50% or 25%. 400–600 mW (50%) 200–300 mW (25%)	No side effects	Improved (*p* < 0.05) 50% power group: baseline BCVA 0.34 ± 0.20 LogMAR End BCVA 0.02 ± 0.13 LogMAR 25% power group: Baseline BCVA 0.27 ± 0.15 LogMAR End BCVA 0.14 ± 0.21 LogMAR	Decreased (*p* < 0.05) 50% power group: Baseline CMT 491.6 ± 154.8 μm End CMT 228.2 ± 88.1 μm at 3 months 25% power group baseline CMT 444.9 ± 164.1 μm End CMT 254.5 ± 101.7 μm at 3 month	3	
(Gawęcki et al., 2019) [77]	2019	Retrospective	32	Symptomatic CSC ≤ 6 months; subfoveal SRF on OCT	577 nm Yellow	160 μm	5%	250 mW	No side effects	Improved (*p* < 0.05) T0: 0.37 ± 0.22 logMAR 6 m 0.22 ± 0.20 logMAR	Decreased (*p* < 0.05) T0: 372.69 ± 89.59 μm 6 m: 262.47 ± 57.74 μm	6	
(Kim et al., 2019) [78]	2019	Retrospective	27	Symptomatic CSC ≥ 3 months; Recurrent CSC and a history of chronic CSC; previous SMYL treatment for CSC; ≥3 years of follow-up data.	577 nm Yellow	100 μm	15%	200–400 mW	No side effects	Improved (*p* < 0.05) T0: 0.26 ± 0.24 LogMAR 3Y: 0.08 ± 0.15 logMAR	Decreased (*p* < 0.05) T0: 389.6 ± 103.4 μm 3Y: 196.4 ± 40.2 μm	3.7 years ± 0.8	
(Gawęcki et al., 2017) [79]	2017	Retrospective	51	Chronic CSCR, visual symptoms ≥ 4 months; no previous retinal laser treatments; absence of CNV	577 nm Yellow	160 μm	5%	250 mW	No side effects	Improved (*p* < 0.05)	Decreased (*p* < 0.05) T0: 301.0 ± 32.1 μm T 2 m: 286.9 ± 21.3 μm	2	
(Ambiya et al., 2016) [80]	2016	Prospective	10	Vision loss > 3 months due to persistent CSC diagnosed by the presence of SRF at the fovea and verified by SD-OCT; focal subfoveal leak on FFA	577 nm yellow laser	100 µm	5%	Laser power titrated to the retinal thermal threshold for each patient; laser power was then reduced to 30% 120–280 mW	No side effects	No statistically significant difference (*p* > 0.05) T0: 73.3 ± 16.1 letters 6 m: 76.9 ± 13.0 letters (*p* = 0.59)	Decreased (*p* < 0.05) T0: 298 ± 128.58 μm 6 m: 214.9 ± 90.10 μm	6	-
(Özmert et al., 2016) [81]	2016	Retrospective	33	CSC with SRF involving the fovea > 6 months; leakage and/or RPE changes on baseline FA; no prior micropulse laser or PDT therapies.	577 nm yellow laser	160 µm	5%	Initially increased upward to the minimum threshold value to cause a barely visible burn on micropulse mode, then reduced by 50%	No side effects	Increased Baseline: 67.3 ± 14.2 ETDRS letters 12 months: 71.5 ± 21.4 ETDRS letters in the SMYL group	Decreased (*p* < 0.05) Baseline: 287.3 ± 126 μm 12 months: 138.0 ± 40 µm in the SMYL group	12	
(Scholz et al., 2016) [82]	2016	Retrospective	100	Persistent SRF owing to cCSC ≥ 6 weeks who were treated with the 577 nm SML or half-dose PDT	577 nm laser	160 μm	5%	Started at 700 mW, then increased stepwise until a just-visible burn appeared and reduced by 50%	No side effects	No statistically significant difference (*p* > 0.05) T0: 0.39 ± 0.24 LogMAR 6 w: 0.31 ± 0.27 LogMAR	Decreased (*p* < 0.05) T0: 445 ± 153 μm 6 w: 297 ± 95 μm	6 weeks	-
(Breukink et al., 2016) [83]	2016	Prospective (prospective treatment protocol consisting of half-dose PDT as a primary treatment in chronic CSC, followed by HSML in nonresponders to two half-dose PDT treatments)	59	Chronic symptomatic CSC with active leakage of fluid under the retina at SD-OCT, ICGA, or/and FA	810 nm diode laser	125 µm	5%	A laser test spot of 1800 mW was first applied. If retinal discoloration was seen at a power of 1800 mW, power was reduced with steps of 300 mW until there was no visible reaction	No side effects	Improvement of BCVA was highest after the first half-dose PDT treatment 0.28 logMAR at baseline to 0.16 logMAR at final follow-up	Decrease of CMT was highest after the first half-dose PDT treatment	6	Patients treated with HSML (n = 10) had previously received 3 mg/m^2^ verteporfin (half-dose) intravenously twice
(Elhamid et al., 2015) [84]	2015	Prospective	15	CSC > 3 months confirmed by FFA and OCT	577 nm laser	200 μm	10%	Laser power titrated to the retinal thermal threshold for each patient, then switched to micropulse mode and power tripled 318 ± 70.63 mW	No side effects	Improved (*p* < 0.05) T0: 0.67 ± 0.097 Snellen 6 m: 0.85 ± 0.097 Snellen	Decreased (*p* < 0.05) T0: 389.6 ± 46.4 µm 6 m: 263.6 ± 24 µm	6	-
(Scholz et al., 2015) [85]	2015	Retrospective	38	Chronic CSC defined by serous SRF on SD-OCT, leakage on FA, and hyperfluorescence on ICG PDT whose treatment ≥3 months prior to SML	577 nm laser	160 µm	5%	Started at 700 mW and then gradually increased until a just-visible burn was seen, then reduced by 50%	No side effects	Improved (*p* < 0.05) T0 0.36 (SD ± 0.24) (logMAR) 6 w 0.33 ± 0.24 3 m 0.27 ± 0.26 6 m 0.29 ± 0.19 T 5.0 ± 3.7 months 0.30 ± 0.25	Decreased (*p* < 0.05) T0 402 (SD ± 139 μm) 6 w 309 ± 86 μm 3 m 263 ± 57 μm 6 m 276 ± 46 μm T 5.0 ± 3.7 months 287 ± 75 μm	5	-
(Yadav et al., 2015) [86]	2015	Case series	15	Patients with idiopathic serous macular detachment on OCT; Leakage at RPE level on FFA or ICGA.	577 nm yellow laser	100 µm	10%	Laser power titrated to the retinal thermal threshold for each patient; laser power was then reduced to 50% from 70 to 200 mW	No side effects	Improved (*p* < 0.05) T0 20/40 (20/20–20/400) T average of 8 weeks (range 4–19 weeks) 20/30	Decreased (*p* < 0.05) average decrease in fluid height was 79%	2 (range 4–19 weeks)	-
(Kim et al., 2015) [87]	2015	Retrospective	10	Symptomatic CSC ≥6; recurrent CSC Patients who had received SMYLP for CSC treatment.	577 nm yellow laser system	100 µm	15%	Laser power titrated to the retinal thermal threshold for each patient; laser power was then reduced to 50% 250–350 mW	No side effects	Improved (*p* < 0.05) T0 0.21 ± 0.21 logMAR 3 m 0.055 ± 0.093 logMAR final follow-up 0.035 ± 0.063 logMAR	Decreased (*p* < 0.05) T0 349.2 ± 53.2 μm 3 m 250.7 ± 28.8 μm final follow-up 261.2 ± 38.31 μm	8 (range 3–18 months)	-
(Malik et al., n.d.) [88]	2015	Retrospective	11 (10 patients)	Diagnosis of CSCR treated with an 810-nm STMP laser Symptomatic CSCR ≥ 3 months	810 nm MicroPulse laser	-	5%	7 patients 950 mW 1 patient 750 mW 1 patient 900 mW 1 patient 1000 mW	No side effects	No statistically significant difference (*p* > 0.05) T0 39.2 letters (SD 15.1) ETDRS 2–12 m 45.5 letters (sd 12) ETDRS	Decreased (*p* < 0.05) T0 414 mm (standard deviation = 137.0 mm) 2–12 m 316.2 mm (standard deviation = 96.5)	2–12	
(Scholz et al., 2015) [85]	2015	Retrospective	38	Serous SRF on SD-OCT, leakage on FA with equivalent hyperfluorescence on ICGA. SRF ≥ 6 weeks Patients treated with PDT whose treatment ≥ 3 months prior to SML.	577 nm Yellow	160 μm	5%	Power titration started at 700 mW and increased until a visible burn. When this threshold was reached, the power was reduced by 50%.	No side effects	Improved (*p* < 0.05) T0 0.36 (SD ± 0.24) (logMAR) 6 w 0.33 ± 0.24 3 m 0.27 ± 0.26 6 m 0.29 ± 0.19 T 5.0 ± 3.7 months 0.30 ± 0.25	Decreased (*p* < 0.05) T0 402 (SD ± 139 μm) 6 w 309 ± 86 μm 3 m 263 ± 57 μm 6 m 276 ± 46 μm T 5.0 ± 3.7 months 287 ± 75 μm	5 ± 3.7	
(Roisman et al., 2013) [89]	2013	Prospective	15	CSC lasting more than 6 months	810 nm diode micropulse laser	125 µm	15%	Laser power titrated to the retinal thermal threshold for each patient; laser power was then increased by 1.2 × threshold 444 mW (range: 300 to 660 mW)	No side effects	Increased T0 35.4 letters (20/49 Snellen equivalent) ± 11.6 3 m 47.9 letters (20/27 Snellen) ± 8.0 6 m 50.0 (20/25 Snellen) ± 6.8	No statistically significant difference (*p* > 0.05) T0 419.6 ± 111.8 mm 3 m 265.4 ± 98.1 mm 6 m 247.2 ± 105.4 mm	6	
(Koss et al., 2012) [90]	2012	Prospective	52	Diagnosis of CSC with no more than two ALSs on FA	810 nm infrared diode laser	125 µm	15%	Once the threshold CW power was determined, the laser was switched from CW into the MicroPulse emission mode, and power was doubled	No side effects	Increased T0 45.4 ± 7.2 TL 6 w 47.8 ± 6.8 TL 6 w 50.5 ± 7.3 TL 10 m 51.6 ± 7.0 TL	Decreased (*p* < 0.05) T0 419 ± 59 mm 6 w 387 ± 94 mm 6 m 329 ± 69 mm 10 m 325 ± 93 mm	10	
(Ricci et al., 2009) [91]	2009	Prospective	7	Idiopathic chronic CSC	810 nm infrared diode laser	112.5 μm	10%	A test burn was performed either in the nasal mid periphery or on a peripheral active leakage site when present to ensure that no visible or latent retinal burn 500 mW power	No side effects	Increased	Increased mean VA improvement at the 12-month follow-up visit was 0.19 LogMAR	from 6 to 12 months	
(Chen et al., 2008) [92]	2008	Prospective	26	CSC ≥ 4 months, with juxtafoveal leakage on FA. Group 1: point source leakage but without associated RPE atrophy. Group 2: point source leakage and associated RPE atrophy. Group 3: diffuse RPE decompensation without definite point source leakage	810 nm diode laser	125 μm	15%	Laser power titrated to the retinal thermal threshold for each patient, then switched to micropulse mode 534.65 ± 179.97 mW	No side effects	Improved (*p* < 0.05) from 0.348 ± 0.228 to 0.124 ± 0.178 after Surgery (logMAR)	Decreased (*p* < 0.05) Mean foveal thickness improved from 340.50 ± 95.34 mm before surgery to 160.53 ± 73.51 mm after surgery	9.5 ± 2.6	
(Lanzetta et al., n.d.) [1]	2008	Prospective	24	Diagnosis of CSC was based on clinical and angiographic patterns	Micropulse diode infrared laser	200 µm	15%	Laser power titrated to the retinal thermal threshold for each patient, then switched to micropulse mode with power from 1 to 2 W (mean, 1.35 W)	No side effects	Increased T0 20/32 (range 20/100–20/20) T final follow-up 20/25 (range 20/200–20/20)	Decreased (*p* < 0.05) T0 328 µm T1M 197 µm T final follow-up 168 µm	14 (range, 3–36 months)	

**Table 3:** SMPL therapy in BRVO-associated edema.

First Author	Year	Study Design	Eyes (N=)	Inclusion Criteria	Laser Type	Spot Diameter	Duty Cycle	Power	Safety	BCVA	CMT	FU (Months)	Additional Treatment
(Feng et al., 2023) [93]	2023	Prospective	94	BRVO with history of ≥3 intravitreal Ranibizumab injections CMT > 250 µm; FFA showing no signs of ischemia	577 nm yellow laser	200 μm	5%	Continuous-wave mode after titrating for each eye	No side effects	Improved (*p* < 0.05) T0: 0.76 ± 0.18 logMAR 6 m: 0.43 ± 0.11 logMAR 12 m: 0.30 ± 0.10 logMAR	Decreased (*p* < 0.05) T0: 490.53 ± 109.27 μm 6 m: 267.56 ± 30.69 μm 12 m: 263.84 ± 33.955 μm	12	
(Terashima et al., 2019) [94]	2019	Retrospective	46	BCVA from 20/400 to 20/25 (Snellen); CRT > 250 μm	577 nm yellow laser	100 μm	15%	Power titrated from 80 mW upward until a burn became visible, then from the continuous wave to the micropulse mode	No side effects	Improved (*p* < 0.05) T0: 0.45 ± 0.24 logMAR 6 m: 0.11 ± 0.15 logMAR	Decreased (*p* < 0.05) T0: 515 ± 172 μm 6 m: 296 ± 98 μm	6	Combination therapy with IVI Ranibizumab + SMPL
(Buyru Özkurt et al., 2018) [95]	2018	Retrospective	51	BRVO ≥ 3 months CMT ≥ 250 μm; BCVA was between 0.22 and 1 (LogMAR),	577 nm yellow laser	100 μm	10%	Power titrated upward until a burn became visible, then from the continuous wave to the micropulse mode	No side effects	No statistically significant difference (*p* > 0.05) Improved (*p* > 0.05) T0: 0.5 ± 0.18 logMAR 6 m: 0.28 ± 0.1 logMAR 12 m: 0.33 ± 0.11 logMAR	Decreased (*p* < 0.05) T0: 495.83 ± 97.74 μm 6 m: 296.5 ± 52.09 μm 12 m: 317.17 ± 37.42 μm	12	
(Parodi et al., 2015) [96]	2015	Prospective	35	Previous conventional grid laser photocoagulation with documented resolution of ME and subsequent recurrence of ME; BCVA between 20/400 and 20/40 CFT ≥ 250 μm	infrared diode laser	125 μm	15%	Power titrated upward until a burn became visible, then continuous wave to the micropulse mode	No side effects	No statistically significant difference (*p* > 0.05) T0: 0.92 +/− 0.31 LogMAR 12 m: 0.99 +/− 0.2 LogMAR	No statistically significant difference (*p* > 0.05) T0: 485.5 +/− 87 μm 12 m: 445 μm	12	
(Inagaki et al., 2014) [97]	2014	Case series	32	BRVO with CMT < 600 μm	810 nm diode laser	200 μm	15%	The lowest energy required to make a visible burn, then used at 60%–90% of that energy level in micropulse mode In 13 eyes 878.46 ± 215.05 mW (750 mW to 1500 mW) In 19 eyes 933.68 ± 417.81 mW (360 mW to 2000 mW)	No side effects	Improved (*p* < 0.05) T0: 0.34 logMAR 12 m: 0.29 logMAR	Decreased (*p* < 0.05) T0: 390.16 ± 94.95 μm 12 m: 312.28 ± 104.90 μm	12	All patients completed 3 months of follow-up, after which additional SMDLP was performed in 11 eyes
(Parodi et al., 2008) [98]	2008	Prospective	24	BRVO occurring 3–18 months earlier; perfused macular edema involving the fovea; BCVA 20/40 or poorer	Infrared diode laser	125 µm	15%	Power was determined by means of a single test burn which brought about a ‘‘medium’’ white burn in continuous wave	No side effects	No statistically significant difference (*p* > 0.05) T0: 0.76 ± 0.34 logMAR 12 m: 0.65 logMAR	Decreased (*p* < 0.05) T0: 429 μm 12 m: 278 μm	12	
(Parodi et al., 2006) [99]	2006	Prospective	36	BRVO occurring 3 to 18 months earlier; macular edema involving the fovea; BCVA 20/40 or poorer	infrared diode laser	125 µm	10%	Power was determined by means of a single test burn which brought about a ‘‘medium’’ white burn in continuous wave 1401.7 ± 171.8 mW (range, 1130–1850)	No side effects	Increased No statistically significant difference (*p* > 0.05) T0: 0.7 logMAR 12 m 0.49 logMAR	Decreased (*p* < 0.05) Decreased (*p* < 0.05) T0: 80 ± 70.7 μm 12 m: 217 ± 50.5 μm	12	
